# Embolo/sclerotherapy for the treatment of hand arteriovenous malformations: a single-center retrospective cohort experience

**DOI:** 10.3389/fsurg.2023.1191876

**Published:** 2023-06-16

**Authors:** Xueqiang Fan, Jianbin Zhang, Peng Liu, Zhidong Ye

**Affiliations:** Department of Cardiovascular Surgery, China–Japan Friendship Hospital (Institute of Clinical Medical Sciences), Chinese Academy of Medical Sciences & Peking Union Medical College, Beijing, China

**Keywords:** arteriovenous malformation (AVM), embolo/sclerotherapy, embolization, sclerotherapy, vascular anomalies

## Abstract

**Purpose:**

To retrospectively report our preliminary experience of treating hand arteriovenous malformations (AVMs) with embolo/sclerotherapy.

**Materials and methods:**

Retrospectively review the demographics, treatment detail, outcome data, and complications of 13 consecutive patients with hand AVMs from January 2018 to December 2021. We embolize the dominant outflow vein with elastic coils and then use absolute ethanol or polidocanol for intravascular sclerotherapy and bleomycin for interstitial sclerotherapy.

**Results:**

Yakes type II presents in four lesions, type IIIa in six, and type IIIb in three. A total of 29 treatment episodes were conducted for the 13 patients (1 episode for 3 patients, 2 for 4 patients, and 3 for 6 patients; the repeated treatment rate was 76.9%). The mean stretched length of coils for 1 treatment episode was 95 cm. The mean absolute ethanol dosage was 6.8 ml (range 4–30 ml). In addition, 10 ml of 3% polidocanol foam was injected and interstitial sclerotherapy with 150,000 IU bleomycin was performed on every patient. The post-operative arterial-dominant outflow vein pressure index (AVI) increased in the 29 procedures (6.55 ± 1.68 vs. 9.38 ± 2.80, *P* < 0.05). The Mann–Whitney *U* test showed that the post-operative AVI was higher in patients without re-intervention (*P* < 0.05). Local swelling occurred after all the procedures. Blistering occurred in 6 of the patients in 13 (44.8%) of the 29 procedures. Superficial skin necrosis occurred in 3 of the patients in 5 (17.2%) of the 29 procedures. The swelling, blistering, and superficial skin necrosis recovered within 4 weeks. No finger amputation occurred. The follow-up time was 6 months. The 6-month assessment of clinical improvement after the last treatment episode showed that 2 patients were cured, 10 were improved, and 1 remained unchanged. With regard to angiographic evaluation, 9 showed partial response and 4 complete response.

**Conclusion:**

Embolo/sclerotherapy can be effective and safe for hand AVM. The AVI increased significantly after embolo/sclerotherapy, and the index may be valuable in predicting recurrence in further study.

## Introduction

Arteriovenous malformations (AVMs) are characterized by direct communications between primitive reticular networks of dysplastic vessels and high-velocity blood shunting from the arterial side to the low-resistance venous side (1). Hand AVMs usually cause more disability or pain than AVMs in other locations because of the elaborate movement and keen sense (2–5).

Determining a treatment strategy for hand AVMs is hard, considering function maintenance and the high complication rate. Partial or sometimes complete excision, embolo/sclerotherapy, and amputation were reported, and the results vary (6–8). Due to its minimally invasive and repeatable nature, embolo/sclerotherapy has emerged as a promising treatment modality for hand AVMs. The Cho–Do and Yakes classification provides interventional therapeutic implications for a specific subset of AVMs (7, 9). However, data on embolo/sclerotherapy for hand AVMs are still lacking.

Starting the year 2018, we have tried treating hand AVMs with embolo/sclerotherapy, which refers to embolizing the dominant outflow vein with elastic coils, then using absolute ethanol or polidocanol for intravascular sclerotherapy and bleomycin for interstitial sclerotherapy. The aim of this study is to retrospectively report our preliminary experience of treating hand AVMs with embolo/sclerotherapy.

## Materials and methods

### Study design

A retrospective cohort study was conducted on 13 consecutive hand AVM patients treated with embolo/sclerotherapy at our institution from January 2018 to December 2021. Patients were identified from the medical record system. The demographics, clinical features, treatment strategy, and prognosis data were extracted and analyzed. The study procedures were in accordance with institutional guidelines. All data were retrospectively collected. The institutional review board does not require approval for the type of research performed. The principles of the Declaration of Helsinki were followed during the study. All patients provided their written informed consent to undergo embolo/sclerotherapy.

### Patient selection

The diagnostic workup for hand AVMs consists of ultrasound, computed tomography angiography, magnetic resonance imaging, and digital subtraction angiography (DSA). The inclusion criteria include (1) patients with obvious or recurrent symptoms, including pain, swelling, discoloration, or ulceration, and (2) diagnostic workup showing that the lesions match the characteristics of hand AVMs.

Patients with severe limb necrosis or uncontrolled infection which is not suitable for embolo/sclerotherapy were excluded.

### Interventional procedure

**Figure 1:**
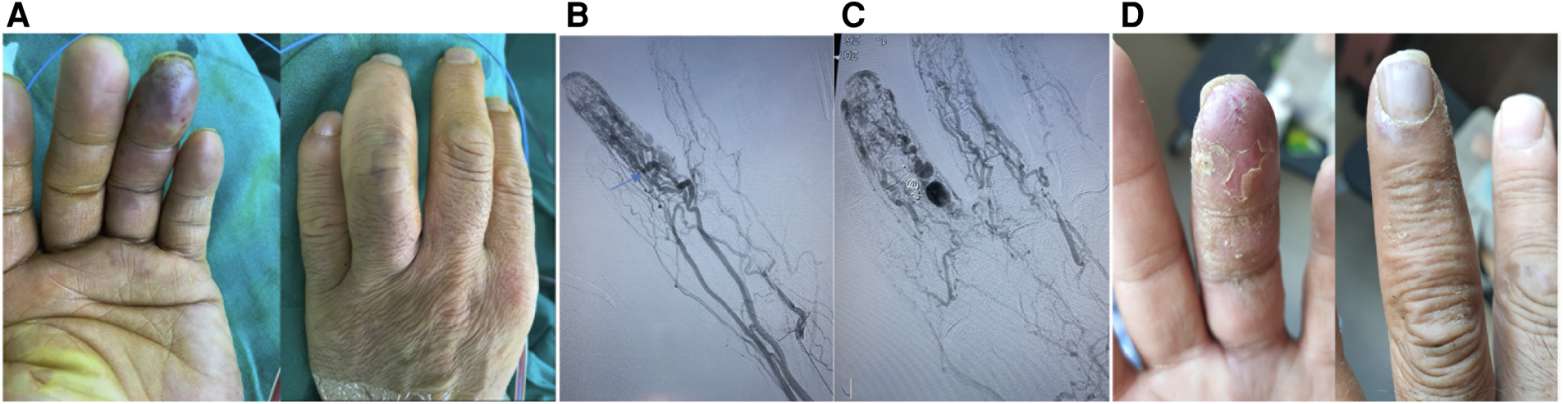
A typical case. This is a 35-year-old male patient whose main complaint was intermittent hemorrhage in the left ring fingertip. (**A**) Abnormal appearance of left ring fingertip; (**B**) pre-operative angiography showed dilation of interphalangeal artery with one dominant outflow vein (Yakes type IIa, Cho–Do type II). The arrow showed the dominant outflow vein. (**C**) Post-operative angiography showed that the fingertip perfusion was better than before; (**D**) 1 month after treatment.

**Figure 2:**
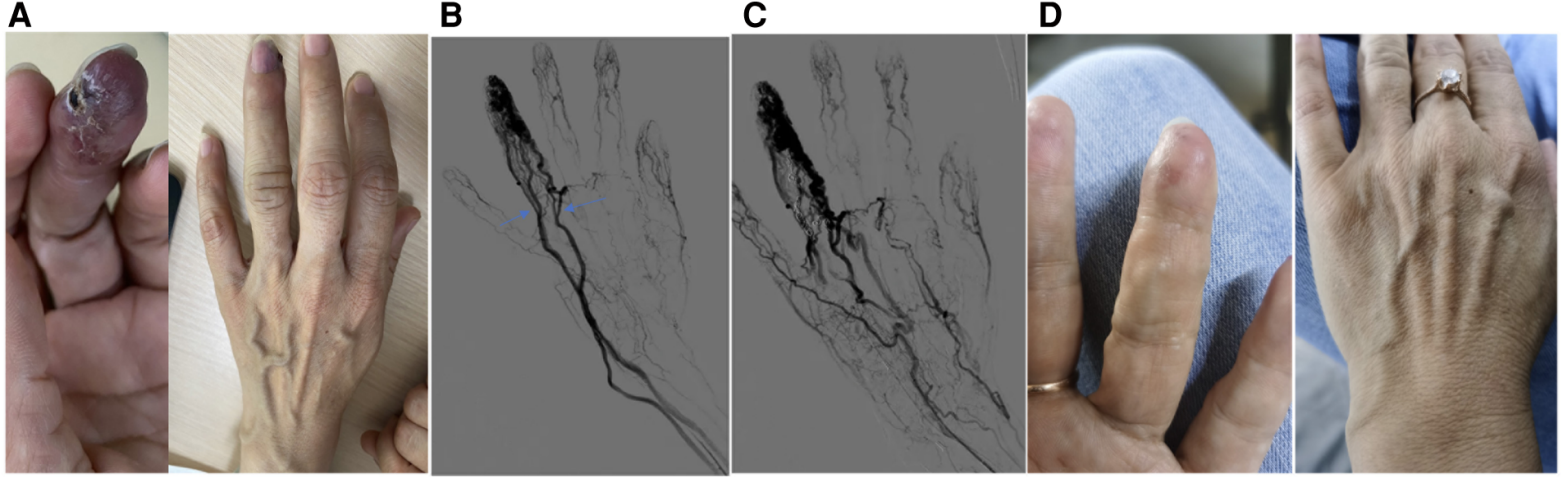
A typical case. This is a 37-year-old female patient whose main complaint was intermittent hemorrhage and pigmentation in the left ring fingertip. (**A**) Abnormal appearance of left ring fingertip and the outflow vein dilation; (**B**) pre-operative angiography showed dilation of interphalangeal artery with multiple dominant outflow vein (Yakes type IIIb, Cho–Do type IIIb). The arrow showed the dominant outflow vein. (**C**) Post-operative angiography showed the fingertip perfusion was better than before: (**D**) 8 months after treatment.

The patients were categorized according to symptoms using the Schöbinger classification. The interventional procedure was reported in detail in Supplementary File. AVMs were categorized according to the DSA feature using the Yakes and Cho–Do classification (7, 9). The polidocanol foam was prepared using the Tessari method (10). Figures 1, 2 show typical cases.

### Arterial-dominant outflow vein pressure index

We routinely measure the pre- and post-operative pressure of the feeding artery and dominant outflow vein. Then, the arterial-dominant outflow vein pressure index (AVI) was calculated by dividing the dominant outflow vein pressure by the feeding artery pressure.

### Endpoints and follow-up

Repeated treatment was necessary for some patients. Clinical improvement (cured, improved, unchanged, and worsened) was assessed and recorded 6 months after the last treatment episode. Angiography after the last treatment episode was evaluated and graded: (1) adverse response means AVM became larger; (2) no response means AVM was ≤50% resolved; (3) partial response means AVM was 50%–99% resolved; and (4) complete response means AVM was 100% resolved. Peri-operative complication was also recorded for those patients.

### Statistical analysis

Continuous variables were presented as mean and standard deviation, or median and interquartile range. Discrete variables were presented as percentages. The Mann–Whitney *U* test was performed to compare continuous variables, and the *χ*^2^ test or Fisher's exact test was performed to compare discrete variables. The Mann–Whitney *U* test was used to test the difference of AVI in patients with or without re-intervention. Data analysis was performed using SPSS version 22 (SPSS Inc., Chicago, IL, United States). A *P*-value of <0.05 was considered statistically significant.

## Results

### Patient characteristics

The final study population consisted of 13 patients (4 men, mean age of 25.86 ± 8.44 years), of which 4 were Schöbinger (9) stage II and 9 were Schöbinger stage III. A total of 29 treatment episodes were performed for the 13 patients (1 episode for 3 patients, 2 for 4 patients, and 3 for 6 patients). Table 1 shows the demographics and clinical features.

**Table 1:** Demographics and clinical features of the patients.

	No. (%)
Age	25.86 ± 8.44 (13–38)
Male	4/7
Symptom	
Pain	10
Ulceration	6
Discoloration	11
Bleeding	4
Functional limitation	11
Thrill and pulsation	9
Location	
Finger	3
Palm	6
Finger + palm	4
Involvement	
Subcutaneous	13
Muscle	5
Bone	1
Yakes classification	
II	4
IIIa	6
IIIb	3
Schöbinger classification	
II	4
III	9
Cho–Do classification	
II	2
IIIa	3
IIIb	8

### Angiographic findings

Arterial angiography was performed through the femoral approach. The feeding arteries were the ulnar and radial arteries. According to the angiographic findings and Yakes classification criteria, type II presents in four lesions, type IIIa in six, and type IIIb in three.

### Dominant outflow vein embolization

Direct puncture to the dominant outflow vein with tourniquet assistance or ultrasound guidance was conducted to establish the embolization approach. In Yakes type II and IIIb patients, multiple outflow veins were embolized. In total, 18 embolization episodes were performed for 13 patients (range 1–4; mean 2.23). A total of 37 detachable coils and 89 pushable coils were used. The mean stretched length of coils for one treatment episode was 95 cm.

### Sclerotherapy

In all 29 treatment episodes, sclerotherapy was performed simultaneously. Absolute ethanol and 3% polidocanol foam were injected when angiography confirmed that the dominant outflow vein flow rate was reduced. Bleomycin was used for interstitial sclerotherapy.

The mean absolute ethanol dosage was 6.8 ml (range 4–30 ml); the 3% polidocanol foam dosage was 10 ml for every patient. Interstitial sclerotherapy with 150,000 IU bleomycin was performed for every patient.

### Clinical and angiographic improvement

The 6-month assessment of clinical improvement after the last treatment episode showed that 2 patients were cured, 10 were improved, and 1 remained unchanged. As far as angiographic evaluation is concerned, 9 showed partial response and 4 showed complete response.

### Complications

No pulmonary hypertension or cardiovascular events occurred. Transient hemoglobinuria occurred in 2 of the patients in 3 (10.34%) of the 29 procedures, of which the absolute ethanol volume was all over 20 ml.

Local swelling occurred after all 29 procedures. Blistering occurred in 6 of the patients in 13 (44.8%) of the 29 procedures. Superficial skin necrosis occurred in 3 of the patients in 5 (17.2%) of the 29 procedures. All swelling, blistering, and superficial skin necrosis recovered within 4 weeks. No finger amputation occurred.

### Arterial-dominant outflow vein pressure index

The post-operative AVI increased in the 29 procedures (6.55 ± 1.68 vs. 9.38 ± 2.80, *P* < 0.05). The Mann–Whitney *U* test showed that the post-operative AVI was higher in patients without re-intervention (*P* < 0.05). However, the multifactor logistic regression analysis did not identify AVI as a predictor for repeated treatment.

## Discussion

The treatment of hand AVMs remains challenging because of the intricate function and rich neurovascular network of the hand. Al-Qattan et al. (8) reported a classification system considering the clinical presentation and anatomical structure to direct the treatment algorithm. Surgical excision is suitable for some patients (11). However, radical excision is frequently impossible and sometimes associated with poor outcomes, recurrences, and complications (12).

It is increasingly recognized that a radical cure for hand AVMs is frequently impossible, and multiple treatment sessions may be required to relieve symptoms (13). Interventional treatment, including embolization and sclerotherapy, appears to be minimally invasive and repeatable. The perception of interventional therapy is also gradually developing. Primitive merely trans-arterial elastic coil embolization without nidus eradication will lead to early recurrence. The concept of curative embolization was first proposed in treating brain AVMs (14). The development of super-selective angiography, liquid embolic agents, and transvenous embolization concepts has facilitated curative embolization. The drainage vein and nidus embolization should be emphasized because mere outflow vein embolization exacerbates venous pressure. The goal of transvenous embolization is to achieve complete nidus filling after drainage vein occlusion (15, 16). Compared with tourniquet interception, coils can achieve permanent embolization to reduce the recurrence rate. Direct puncture to the venous sac could also be used when the transvenous approach is not feasible. In fact, direct puncture was most commonly used in our study series, maybe due to the smaller diameter and tortuousness of the hand vessel.

After solid embolic agent embolization to reduce the blood flow rate, a liquid sclerosant to eradicate nidus is also important. Absolute ethanol has been proven as a potent sclerosant to thoroughly destroy the endothelium of the nidus, which causes denaturation of serum protein, decrease in oxygen tension, and release of angiogenic factors (17, 18). Ethanol embolization could achieve complete symptom relief and nidus devascularization (19). However, the ectopic embolism and skin necrosis rate is also relatively high for absolute ethanol (19). Inevitable ectopic embolism seems likely to occur because of the limited region and intimate relationship between normal and abnormal vessels in the hand, which might be the reason why the cure rate in the hand is lower than that in other locations (20). Therefore, we sometimes use 3% polidocanol foam to replace absolute ethanol. When we cannot confirm whether the puncture needle is in an abnormal vessel or worry about sclerosant leakage, injecting intralesional interstitial bleomycin is a safe and effective choice (21). The total cumulative dose of less than 400,000 IU bleomycin was recommended in the case of pulmonary interstitial fibrosis. The bleomycin polidocanol form has also been reported as an effective sclerosant (22).

Normally, arterial pressure is much higher than venous pressure. In AVM patients, the blood flow in the outflow vein increases significantly, which increases the pressure in the outflow vein. We calculated the AVI by dividing the dominant outflow vein pressure by the feeding artery pressure. After embolo/sclerotherapy, the arterial pressure might be increased because the peripheral resistance was increased, and the dominant outflow vein pressure might be decreased because the blood flow decreased. In our data, the AVI in most of the patients increased after treatment. In patients without re-intervention, the post-operative AVI was higher, but further analysis did not identify AVI as a predictor of repeated treatment. The AVI is just a preliminary analysis and was not reported before; whether this index is helpful needs further investigation.

Several limitations may exist in this study. First, the sample size is relatively small because hand AVM is a rare disease. Second, this is only our preliminary retrospective experience; the evaluation of clinical improvement is too subjective. Third, some patients in the study series still need further treatment in the future and the follow-up time is short, so this might not be the final result.

## Conclusion

In conclusion, embolo/sclerotherapy can be effective and safe for hand AVM. The AVI increased significantly after embolo/sclerotherapy, and the index may be valuable in predicting recurrence in further study.

## Data Availability

The original contributions presented in the study are included in the article/**Supplementary Material**, further inquiries can be directed to the corresponding author.
