# Supplementary material for: Embolo/sclerotherapy for the treatment of hand arteriovenous malformations: a single-center retrospective cohort experience
Source: Front Surg. 2023 Jun 16;10:1191876. doi: 10.3389/fsurg.2023.1191876 (PMC10312000; doi:10.3389/fsurg.2023.1191876)
Supplement: Supplementary file 1 [file Table1.docx]

Supplementary material: Schöbinger, Yakes and Cho-Do classification

| Stage | Features |
| --- | --- |
| Stage I: Quiescence | May or may not have a vascular skin stain, warmth of the affected tissues, and AV shunts can be detected by Doppler US scanning. The AVM is present but causes no clinical symptoms |
| Stage II: Expansion of the AVM lesion | Stage I plus enlargement, pulsations, palpable thrill, audible bruit and enlarged arterialized tortuous/tense veins |
| Stage III: Destructive tissue changes | Stage II plus dystrophic skin changes, skin ulcerations that can be nonhealing, bleeding from the ulcerated areas in the skin or mucosal surfaces, overt tissue necrosis, and lytic lesions of bone may occur |
| Stage IV: Decompensation | Stage III plus congestive cardiac failure with increased cardiac output, abnormally lowered PVR, and venous hypertension secondary tissue and skin changes |

Table-1: Schöbinger classification for AVM symptomatology

AV arteriovenous, AVM arteriovenous malformation, PVR peripheral vascular resistance, US ultrasound.

| Classification | Features |
| --- | --- |
| I | A direct artery/arteriole to vein/venule connection |
| IIa型 | Multiple inflow arteries into a “nidus” pattern with direct artery-arteriolar to vein-venular structures that not be aneurysmal |
| IIb型 | Multiple inflow arteries into a “nidus” pattern with direct artery-arteriolar to vein-venular structures that be aneurysmal, the nidus is before the aneurysmal vein |
| IIIa | Multiple inflow arterioles shunting into an aneurysmal vein that has a single vein outflow |
| IIIb | Multiple inflow arterioles shunting into an aneurysmal vein with multiple outflow veins |
| IV型 | Multiple arteries/arterioles which form innumerable microfistulae that diffusely infiltrate the affected tissue |

Table-2: Yakes classification for AVM

| Classification | Features |
| --- | --- |
| I | Arteriovenous fistulae: no more than 3 separate arteries shunt to the initial part of a single venous component. |
| II | Arteriolovenous fistulae: multiple arterioles shunt to the initial part of a single venous component, in which the arterial components show a plexiform appearance on angiography |
| IIIa | Arteriolovenulous fistulae with non-dilated fistula: fine multiple shunts are present between arterioles and venules and appear as a blush or fine striation on angiography. |
| IIIb | Arteriolovenulous fistulae with dilated fistula: multiple shunts are present between arterioles and venules and appear as a complex vascular network on angiography. |

Table-3: Cho Do classification for AVM
