# Supplementary material for: Embolo/sclerotherapy for the treatment of hand arteriovenous malformations: a single-center retrospective cohort experience
Source: Front Surg. 2023 Jun 16;10:1191876. doi: 10.3389/fsurg.2023.1191876 (PMC10312000; doi:10.3389/fsurg.2023.1191876)
Supplement: Supplementary file 2 [file Table2.docx]

Supplementary material: Interventional procedure

All the embolo/sclerotherapy procedures were carried out under general anesthesia. Patients were in supine position with abduction of the affected upper limb. Continuous arterial pressure was monitored through invasive contralateral radial artery access. Pulmonary artery pressure was not routinely monitored. Pre-operative trans-arterial angiography was carried out routinely and AVMs were categorized by Yakes and Cho Do classification^[7,9]^ according to the DSA feature. Dominant outflow vein was defined as the vein with maximum diameter and fastest flow rate. When dominant outflow vein was determined, we retrogradely punctured the dominant outflow vein with a 21-Gauge micro puncture needle (Micropuncture Check-Flo Performer Introducer Set, Cook) and exchanged the sheath, then advanced a 2.4F microcatheter (Renegade STC 18, Boston Scientific) as close as possible to the nidus (the nidus indicate the lesion that allow high-pressure arterial blood shunts directly into the venous side, without an intervening capillary bed). Detachable coil (Interlock, Boston Scientific) and pushable coil (Tornado, Cook) was used for dominant outflow vein embolization. The type, size, number, and total length of elastic coil was recorded.

When angiography confirmed that the blood flow rate in dominant outflow vein was obviously reduced, the sclerotherapy was then carried out. A 21-Gauge micro puncture needle or a 22-Gauge scalp needle (Hanaco, China) was used to puncture the nidus. If blood backflow was good and angiography confirmed the needle was in nidus, absolute ethanol or polidocanol foam was used (the concentration of polidocanol is 3% and foam was prepared with Tessari method^[10]^, dilution ratio to air was 1:4). The limited dosage was < 1mL/kg for absolute ethanol and < 2mg/kg for polidocanol. When the needle was not in vessel, we used bleomycin for interstitial sclerotherapy (150,000 IU bleomycin was dissolved in 10 milliliter 0.9% sodium chloride solution, mixed with 5 mg dexamethasone and 100 mg lidocaine to prepare the bleomycin solution).
