# Supplementary material for: Embolo/sclerotherapy for the treatment of hand arteriovenous malformations: a single-center retrospective cohort experience
Source: Front Surg. 2023 Jun 16;10:1191876. doi: 10.3389/fsurg.2023.1191876 (PMC10312000; doi:10.3389/fsurg.2023.1191876)
Supplement: Supplementary file 3 [file Table3.docx]

Supplementary material: previous reports on hand avms and their outcomes

|  | n |  |
| --- | --- | --- |
| Li et al, 2019 | 12 | Ethanol embolization has the potential to control high-flow hand AVMs by using coil-assisted DOV occlusion with an acceptable risk of minor and major risks. |
| Park et al, 2010 | 31 | Ethanol embolotherapy of hand AVMs improves symptoms in a certain percentage of patients with a relatively high risk of complications. According to the extent of AVMs, there was a trend toward a higher complication rate in treatment of AVMs involving fingers and a lower rate of therapeutic benefit in AVMs involving both the finger and the palm. |
| Park et al, 2012 | 64, including 41 Embolo/sclerotherapy | AVM treatment, and especially embolo/sclerotherapy, is a long-term prospect, and it carries a potential risk for serious complications. After every treatment, the lesions must be reevaluated and new treatment plans must be made by the members of a multidisciplinary team. |
