# Supplementary material for: Embolo/sclerotherapy for the treatment of hand arteriovenous malformations: a single-center retrospective cohort experience
Source: Front Surg. 2023 Jun 16;10:1191876. doi: 10.3389/fsurg.2023.1191876 (PMC10312000; doi:10.3389/fsurg.2023.1191876)
Supplement: Supplementary file 4 [file Presentation1.pptx]

## Slide 1
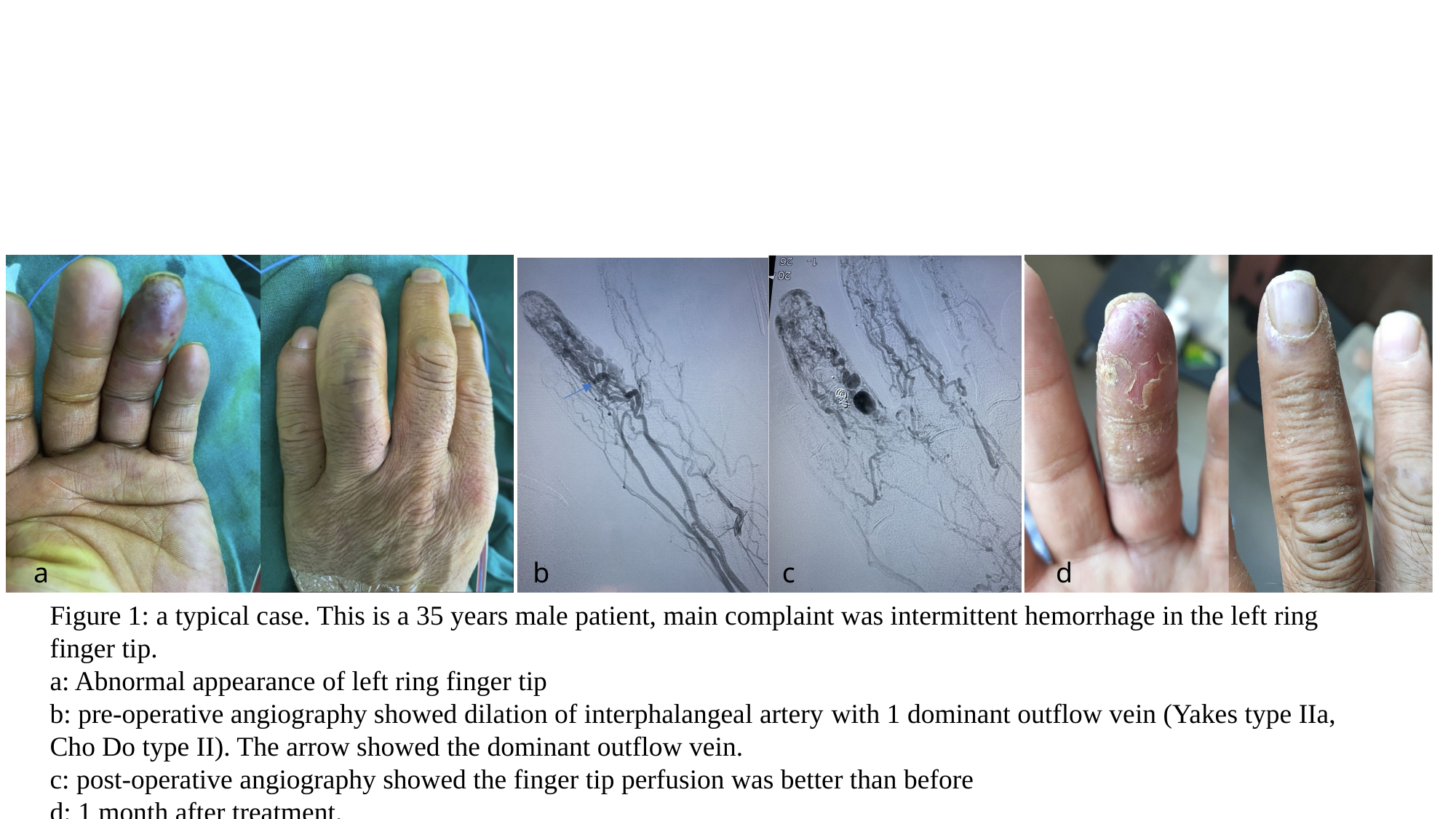

c
d
a
b
Figure 1: a typical case. This is a 35 years male patient, main complaint was intermittent hemorrhage in the left ring finger tip.
a: Abnormal appearance of left ring finger tip
b: pre-operative angiography showed dilation of interphalangeal artery with 1 dominant outflow vein (Yakes type IIa, Cho Do type II). The arrow showed the dominant outflow vein.
c: post-operative angiography showed the finger tip perfusion was better than before
d: 1 month after treatment.

## Slide 2
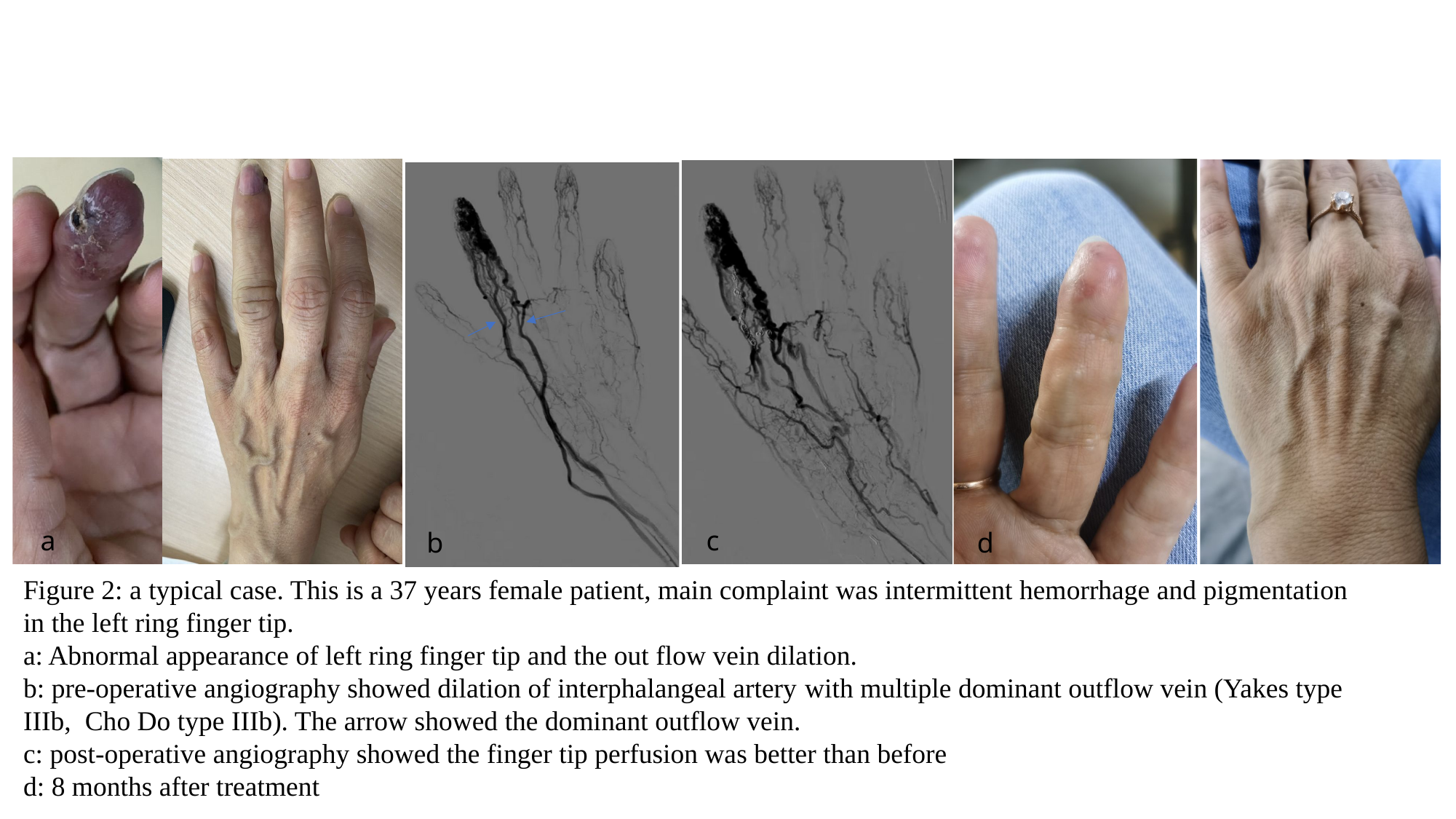

a
c
b
d
Figure 2: a typical case. This is a 37 years female patient, main complaint was intermittent hemorrhage and pigmentation in the left ring finger tip.
a: Abnormal appearance of left ring finger tip and the out flow vein dilation.
b: pre-operative angiography showed dilation of interphalangeal artery with multiple dominant outflow vein (Yakes type IIIb, Cho Do type IIIb). The arrow showed the dominant outflow vein.
c: post-operative angiography showed the finger tip perfusion was better than before
d: 8 months after treatment
